# Supplementary material for: Antibodies targeting ADAM17 reverse neurite outgrowth inhibition by myelin-associated inhibitors
Source: Life Sci Alliance. 2025 Mar 25;8(6):e202403126. doi: 10.26508/lsa.202403126 (PMC11938383; doi:10.26508/lsa.202403126)
Supplement: Supplementary file 1 [file LSA-2024-03126_TableS1.docx]

**Supplementary Table S1.**

| no MAG | | MAG | D8P1C1+MAG | D5P2A11+MAG | | | C12+MAG | | TAPI+MAG | | Bati+MAG | | GI+MAG |
| --- | --- | --- | --- | --- | --- | --- | --- | --- | --- | --- | --- | --- | --- |
| 86.5 | | 10.1 | 53 | 55 | | | 38 | | 36.8 | | 12.8 | | 8 |
| 83.7 | | 12.3 | 56.8 | 48 | | | 34.4 | | 38.3 | | 13.8 | | 6 |
| 78.6 | | 16 | 67.4 | 52 | | | 35.8 | | 37.9 | | 12.7 | | 7 |
| 75 | | 11.4 | 66.9 | 54 | | | 33 | | 31 | | 11.6 | | 8 |
| 82.4 | | 17.3 | 62.1 | 51 | | | 31.5 | | 28 | | 11 | | 10 |
| 86.5 | | 12.5 | 50.8 | 67 | | | 30 | | 29.6 | | 13 | | 12 |
| 80.4 | | 13.1 | 65 | 68.3 | | | 29.5 | | 33 | | 15 | | 13 |
| 82 | | 12 | 59 | 67.9 | | | 33.4 | | 38 | | 16 | | 12 |
| 81 | | 13 | 45 | 66.3 | | | 36.7 | | 41 | | 17 | | 11 |
| 78 | | 18 | 64 | 52.1 | | | 32.8 | | 42.9 | | 12.6 | | 16 |
| 85.5 | | 19.2 | 63 | 53 | | | 30.6 | | 40.6 | | 10.5 | | 15 |
| 76 | | 11 | 54 | 52 | | | 29.7 | | 38.9 | | 11.7 | | 17 |
| 76 | | 8 | 55.8 | 50.5 | | | 33 | | 36.8 | | 15.4 | | 12 |
| 89 | | 7 | 63.2 | 50.3 | | | 37.8 | | 38.9 | | 16.1 | | 10 |
| 75 | | 8.3 | 60.8 | 50.6 | | | 23.8 | | 37.3 | | 12.6 | | 9.7 |
| 74 | | 8.9 | 62.9 | 64.7 | | | 43 | | 32.3 | | 11.8 | | 9.8 |
| 85 | | 12.5 | 59.8 | 66 | | | 35 | | 34.4 | | 11.5 | | 11.7 |
| 83 | | 16.2 | 58.8 | 39.7 | | | 34 | | 30.6 | | 11 | | 12.6 |
| 80 | | 17.4 | 57.6 | 45.6 | | | 27 | | 29.8 | | 13.8 | | 13.8 |
| 80 | | 12.5 | 63.3 | 51.9 | | | 28 | | 36.7 | | 12.5 | | 11.7 |
| 81 | | 11.3 | 64.4 | 50.6 | | | 42 | | 35.4 | | 15 | | 13.6 |
| 77.6 | | 15.5 | 62.8 | 44.4 | | | 44.2 | | 28.9 | | 16 | | 12.8 |
| 84.3 | | 19 | 60.7 | 47.8 | | | 37.8 | | 31.8 | | 14.8 | | 11.5 |
| 79.3 | | 12 | 59.7 | 46.6 | | | 32.2 | | 38 | | 13.8 | | 10.6 |
| 77.6 | | 11.9 | 64.3 | 42.8 | | | 35.6 | | 32 | | 13.9 | | 10.6 |
| 82.8 | | 10.4 | 66.2 | 54.3 | | | 38.8 | | 47 | | 12.7 | | 11.7 |
| 87 | | 9.2 | 67 | 55.1 | | | 37.5 | | 45.8 | | 14.8 | | 12.8 |
| 86 | | 8.2 | 61 | 53.2 | | | 36 | | 30 | | 15.5 | | 13.7 |
| 87.4 | | 7.9 | 58 | 55.1 | | | 27 | | 28 | | 16.8 | | 13.8 |
| 87 | | 13.7 | 52 | 62.7 | | | 37 | | 28 | | 15.8 | | 12.9 |
| 85 | | 12.9 | 43 | 58.5 | | | 41.5 | | 29 | | 12 | | 13.5 |
| 80 | | 11.8 | 68 | 61 | | | 40.5 | | 33 | | 10 | | 14 |
| 73 | | 13.8 | 53 | 60.8 | | | 42.3 | | 38 | | 9 | | 15 |
| 82 | | 19.4 | 55.7 | 62.8 | | | 54.3 | | 31 | | 10.5 | | 16 |
| 84.4 | | 14.5 | 46 | 60.7 | | | 52.6 | | 27 | | 11.7 | | 12 |
| 86.9 | | 13.8 | 44 | 59.8 | | | 34.8 | | 25.7 | | 12.7 | | 9 |
| 82.1 | | 14.9 | 48.9 | 60.4 | | | 33.7 | | 26.9 | | 12.4 | | 16 |
| 79 | | 11.6 | 49.5 | 63.3 | | | 31 | | 24.5 | | 12 | | 16.7 |
| 77.5 | | 14.2 | 56 | 54 | | | 43 | | 28.9 | | 13 | | 14.6 |
| 71 | | 13.7 | 55 | 51 | | | 34.9 | | 31.8 | | 14 | | 14.5 |
| 70 | | 12.9 | 51 | 59 | | | 32 | | 36 | | 12 | | 13.7 |
| 83 | | 10.6 | 59 | 53.3 | | | 30.6 | | 35.6 | | 15 | | 15.7 |
| 84 | | 8.9 | 63 | 52 | | | 35.7 | | 37.8 | | 12 | | 12 |
| 82.3 | | 16.8 | 64 | 57 | | | 34.9 | | 42 | | 14.6 | | 14.7 |
| 81 | | 11.8 | 63 | 58 | | | 33.5 | | 40 | | 13 | | 13.8 |
| 80 | | 12.8 | 47 | 59.3 | | | 29.8 | | 31 | | 14 | | 11.8 |
| 80.5 | | 13.9 | 53 | 44 | | |  | | 44 | | 15 | | 15.8 |
| 72.1 | | 11.1 | 50.5 | 41 | | |  | | 42 | | 13 | | 17.8 |
| 76.9 | | 10.7 | 50.7 | 52 | | |  | | 48 | | 15 | | 12 |
| 75.5 | |  | 430 | 65 | | |  | | 53 | |  | |  |
| 77.3 | |  | 56 | 63 | | |  | | 51 | |  | |  |
| 74.2 | |  | 55.5 | 48 | | |  | | 38 | |  | |  |
| 76.1 | |  | 54.9 | 39 | | |  | | 37.8 | |  | |  |
| 73.3 | |  | 53.2 | 53 | | |  | | 37 | |  | |  |
| 81.7 | |  | 65 | 52.4 | | |  | | 31.1 | |  | |  |
| 80.4 | |  | 63 | 55.8 | | |  | | 38 | |  | |  |
| 81.2 | |  | 60 | 53 | | |  | |  | |  | |  |
| 78.4 | |  | 58.5 | 62.7 | | |  | |  | |  | |  |
| 79.3 | |  |  | 66.8 | | |  | |  | |  | |  |
| 72 | |  |  | 63 | | |  | |  | |  | |  |
| 71 | |  |  | 69 | | |  | |  | |  | |  |
| 69 | |  |  | 61 | | |  | |  | |  | |  |
| 68 | |  |  | 60.8 | | |  | |  | |  | |  |
| 78 | |  |  | 63.8 | | |  | |  | |  | |  |
| 81 | |  |  | 62.8 | | |  | |  | |  | |  |
| 86 | |  |  | 54 | | |  | |  | |  | |  |
| 83 | |  |  | 51 | | |  | |  | |  | |  |
| 82.9 | |  |  |  | | |  | |  | |  | |  |
| 85.6 | |  |  |  | | |  | |  | |  | |  |
| 75 | |  |  |  | | |  | |  | |  | |  |
| 74 | |  |  |  | | |  | |  | |  | |  |
| 73.8 | |  |  |  | | |  | |  | |  | |  |
| 77 | |  |  |  | | |  | |  | |  | |  |
| 72 | |  |  |  | | |  | |  | |  | |  |
|  | |  |  |  | | |  | |  | |  | |  |
|  |  | |  | |  |  | |  | |  | |  |  |

**Table S1. Measured lengths of the longest primary neurites from each cell in microns.**
