# Supplementary material for: Antibodies targeting ADAM17 reverse neurite outgrowth inhibition by myelin-associated inhibitors
Source: Life Sci Alliance. 2025 Mar 25;8(6):e202403126. doi: 10.26508/lsa.202403126 (PMC11938383; doi:10.26508/lsa.202403126)
Supplement: Supplementary file 2 [file LSA-2024-03126_TableS2.docx]

| **Supplementary Table S2.** | | |  |  |  |  |
| --- | --- | --- | --- | --- | --- | --- |
| Sample1-Sample2 | Test Statistic | Std. Error | Std. Test Statistic | Sig. | Adj. Sig.^a^ |  |
| MAG vs GI | -0.786 | 26.098 | -0.030 | 0.976 | 1.000 |  |
| MAG vs Bati | -11.857 | 26.098 | -0.454 | 0.650 | 1.000 |  |
| MAG vs C12 | -130.345 | 26.520 | -4.915 | 0.000 | 0.000 |  |
| MAG vs TAPI-1 | -131.688 | 25.269 | -5.211 | 0.000 | 0.000 |  |
| MAG vs D5P2A11 | -234.304 | 24.282 | -9.649 | 0.000 | 0.000 |  |
| MAG vs D8P1C1 | -246.241 | 25.166 | -9.785 | 0.000 | 0.000 |  |
| MAG vs no MAG | 340.674 | 23.792 | 14.319 | 0.000 | 0.000 |  |
| GI vs C12 | 129.559 | 26.520 | 4.885 | 0.000 | 0.000 |  |
| GI vs TAPI-1 | 130.902 | 25.269 | 5.180 | 0.000 | 0.000 |  |
| GI vs D5P2A11 | 233.518 | 24.282 | 9.617 | 0.000 | 0.000 |  |
| GI vs D8P1C1 | 245.455 | 25.166 | 9.754 | 0.000 | 0.000 |  |
| GI vs no MAG | 339.888 | 23.792 | 14.286 | 0.000 | 0.000 |  |
| Bati vs GI | 11.071 | 26.098 | 0.424 | 0.671 | 1.000 |  |
| Bati vs C12 | 118.488 | 26.520 | 4.468 | 0.000 | 0.000 |  |
| Bati vs TAPI-1 | 119.830 | 25.269 | 4.742 | 0.000 | 0.000 |  |
| Bati vs D5P2A11 | 222.447 | 24.282 | 9.161 | 0.000 | 0.000 |  |
| Bati vs D8P1C1 | 234.383 | 25.166 | 9.314 | 0.000 | 0.000 |  |
| Bati vs no MAG | 328.817 | 23.792 | 13.821 | 0.000 | 0.000 |  |
| C12 vs TAPI-1 | -1.343 | 25.705 | -0.052 | 0.958 | 1.000 |  |
| C12 vs D5P2A11 | 103.959 | 24.735 | 4.203 | 0.000 | 0.001 |  |
| C12 vs D8P1C1 | 115.896 | 25.603 | 4.527 | 0.000 | 0.000 |  |
| C12 vs no MAG | 210.329 | 24.254 | 8.672 | 0.000 | 0.000 |  |
| TAPI-1 vs D5P2A11 | 102.616 | 23.389 | 4.387 | 0.000 | 0.000 |  |
| TAPI-1 vs D8P1C1 | 114.553 | 24.305 | 4.713 | 0.000 | 0.000 |  |
| TAPI-1 vs no MAG | 208.986 | 22.880 | 9.134 | 0.000 | 0.000 |  |
| D5P2A11 vs D8P1C1 | 11.937 | 23.277 | 0.513 | 0.608 | 1.000 |  |
| D5P2A11 vs no MAG | 106.370 | 21.784 | 4.883 | 0.000 | 0.000 |  |
| D8P1C1 vs no MAG | 94.433 | 22.765 | 4.148 | 0.000 | 0.001 |  |

**Table S2. Pairwise comparisons of treatment.** For S2B, each row tests the null hypothesis that the Sample 1 and Sample 2 distributions are the same. Asymptotic significances (2-sided tests) are displayed. The significance level is 0.050. ^a^Significance values have been adjusted by the Bonferroni correction for multiple tests. Both S2A and S2B are supplementary to Figure 7.

Bati: Batimastat, MMP inhibitor; GI: GI254023X, ADAM10 inhibitor; TAP1-1: ADAM17 inhibitor
